# Prevalence and Correlates of Prehypertension and Hypertension among Adults in Northeastern China: A Cross-Sectional Study

**DOI:** 10.3390/ijerph13010082

**Published:** 2015-12-25

**Authors:** Guang Yang, Yue Ma, Shibin Wang, Yingying Su, Wenwang Rao, Yingli Fu, Yaqin Yu, Changgui Kou

**Affiliations:** Department of Epidemiology and Biostatistics, School of Public Health, Jilin University, Changchun 130021, China; 15143086676@163.com (G.Y.); mayue205@163.com (Y.M.); spiriorwang@126.com (S.W.); suyy14@mails.jlu.edu.cn (Y.S.); raoww14@mails.jlu.edu.cn (W.R.); fuyingli318@126.com (Y.F.); yuyaqin5540@163.com (Y.Y.)

**Keywords:** hypertension, prevalence, correlate

## Abstract

*Background*: Prehypertension is a category between normotension and hypertension that is becoming increasingly common in China. However, limited data are available on the prevalence and correlates of prehypertension in northeastern China. *Methods*: A cross-sectional study using stratified, clustered multistage, and random sampling methods was performed on 17,584 participants. *Results*: The prevalence of prehypertension and hypertension was 36.0% and 30.8% in northeastern China, respectively. As age increased, the prevalence of prehypertension in males declined (*p*-trend < 0.001), in parallel to an increase in the prevalence of hypertension (*p*-trend < 0.001). The prevalence of hypertension for females increased as age increased (*p*-trend < 0.001). Logistic regression analysis showed that age, gender, location, drinking, Body Mass Index (BMI), abdominal obesity, hypertriglyceridemia, and hypercholesterolemia correlated with prehypertension and hypertension (*p*-trend < 0.05). *Conclusions*: This study revealed a high prevalence of prehypertension and hypertension in an adult population of northeastern China and some correlates of prehypertension and hypertension.

## 1. Introduction

Hypertension is one of the major risk factors for global mortality and is estimated to have caused 9.4 million deaths, according to the WHO Global Status Report on Non-communicable Diseases in 2014 [1]. The prevalence of hypertension in the adult population varies from 5.2% to 70.7% worldwide, and it is estimated that more than 1.5 billion individuals currently have hypertension [2,3]. To draw attention to the increased risks resulting from elevated blood pressure, the concept of prehypertension has been defined. Prehypertension is a new category between normotension and hypertension and was proposed by the Seventh Report of the Joint National Committee (JNC-7) on Prevention, Detection, Evaluation, and Treatment of High Blood Pressure in 2003 [4]. It is defined as systolic blood pressure (SBP) of 120–139 mmHg and/or diastolic blood pressure (DBP) of 80–89 mmHg in adults 18 years and older. According to the standard of the JNC-7, individuals with prehypertension have a higher risk of developing clinical hypertension than those with ideal blood pressure levels, and they have an increased risk of cardiovascular disease [5,6]. According to the study of M.Fareed *et al*. [7], prehypertension is associated with a 1.7-fold increase in coronary artery disease and a 3.5-fold increase in myocardial infarction.

With the recent rapid economic growth and urbanization of China, the prevalence of hypertension and prehypertension has increased significantly. Although numerous studies have focused on prehypertension, epidemiological data on the prevalence and correlates of prehypertension and hypertension in northeastern China are limited. The aims of the present study were: (1) to estimate the prevalence of prehypertension and hypertension in the Jilin Province in northeastern China; (2) to examine the correlates of prehypertension and hypertension from a population-based study of Chinese adults; and (3) to provide scientific references on the primary prevention, early detection, and intervention strategies for treating prehypertension and hypertension.

## 2. Experimental Section

### 2.1. Subjects

Data were obtained from a large-scale, cross-sectional epidemiological study in Jilin Province in 2012. Community residents who were aged 18–79 years old and had lived in Jilin Province for 6 months were recruited. A sample of 1‰ of the total population was used to obtain a representative sample of the population. A face-to-face questionnaire interview was performed by the investigators who had been uniformly trained, and participants then underwent the anthropometric checks including height and weight.

This process included five steps. First, nine regions were sampled according to the proportion of the population; second, 32 districts or counties were sampled according to rural and urban locations; third, three neighborhood committees were sampled in each counties; fourth, one resident group was sampled in each neighborhood committee; finally, one adult resident was randomly selected from each household of the selected resident groups [8].

We contacted 23,050 people to participate in the survey. Only 17,584 individuals completed both the questionnaire and the anthropometric measurements in the present study, yielding a response rate of 76.3%. This study was conducted in a national comprehensive university located in Jilin Province in northeastern China. Approval from the Ethics Committee of Jilin University School of Public Health (Approval Number: 2012-R-011) was obtained before the study commenced.

### 2.2. Data Collection

The data collection period was from July to August 2012. Participants were asked to complete a questionnaire designed by our project team through face-to-face interviews; trained observers made anthropometric examinations using standardized procedures. Information obtained from the questionnaire included socio-demographic characteristics, lifestyle, and family history. The anthropometric examinations included body weight, height, waist circumference, and hip circumference.

In addition, specially assigned people managed quality control. Before the official investigation, a pre-investigation was conducted to explore the design of the questionnaire. All of the investigators were medical students who were systematically trained. To ensure that the data collected were high-quality and representative, standard protocols and instruments were used, the certification requirements for data collection were strict, and a quality assurance program was conducted.

### 2.3. Blood Pressure Measurement and Classification

Blood pressure was measured using an electric sphygmomanometer (HEM-7200, OMRON, Dalian, China). Two measurements were taken after the participants had rested for at least 5 min. SBP or DBP was defined as the average of the two SBP or DBP readings.

### 2.4. Other Variable Measurements and Classification

Information on socio-demographic characteristics, lifestyle, and family history was self-reported. Socio-demographic characteristics included age, gender, ethnicity, educational status, marital status, occupation and family income *per capita*. Lifestyle included drinking and dietary intake. Dietary intake assessment was done under two headings: (1) Salt intake and (2) Frequency of fruit, egg and meat intakes. “Salt intake” was divided into three levels: light, moderate, and salty. The participants made their own choices based on life experience. Frequencies of eating fruits, eggs or meat were divided into two: Frequently/sometimes and seldom/never. Frequently/sometimes fruits, eggs or meat intake was defined as eating these food ≥2 times/week; while seldom/never was defined as eating <2 times/week. Body weight, height, waist circumference, and hip circumference were measured using a standardized method. Body mass index (BMI) was calculated as weight(kg)/height(m^2^). Waist circumference was measured at the level of the umbilicus. Hip circumference was measured at the level of the greater trochanter. A fasting blood specimen was collected for lipid analysis. Dyslipidemia, including triglycerides and total cholesterol, was based on the cut-off points of 150 mg/dL (1.70 mmol/L) and 200 mg/dL (5.18 mmol/L), respectively [9].

### 2.5. Statistical Analysis

Data were entered into a computer using EpiData (Version 3.1, Odense, Denmark) and were analyzed using SPSS (Version 21.0, IBM SPSS, IBM Corp, Armonk, NY, USA). Continuous variables were expressed using mean and standard deviation. Categorical variables were presented as frequencies. The prevalence (PR) and 95% confidence interval (CI) of prehypertension and hypertension were adjusted by complex weighted computation with the gender, age, and location distribution, according to the Sixth Chinese National Population Census, 2010, and were calculated using the Taylor Series approach. The Rao-Scott χ^2^ test was used to assess differences in categorical variables based on the complex sampling model. Univariate and multivariate logistic regression analyses were used to test the significant determinants of prehypertension *vs.* normotension and hypertension *vs.* prehypertension. Complex weighted computation was used to analyze all of the statistics.

## 3. Results

### 3.1. Prevalence of Prehypertension and Hypertension

There were 8096 males and 9488 females in the survey. The mean age was 47.81 ± 13.20 years (47.10 ± 13.80 years in males, 48.42 ± 12.63 in females). The prevalence of prehypertension and hypertension in the socio-demographic characteristics in the study is reported in Table 1. Based on the complex weights computation, the prevalence of normotension, prehypertension, and hypertension was 33.3%, 36.0%, and 30.8%, respectively. The prevalence of normotension and prehypertension decreased with age, but the prevalence of hypertension increased with age, especially in the ≥ 45 years population. The mean age of normotension was 41.98 ± 12.27 years, and the gender ratio (males: females) was 1:2.24. The mean age of prehypertension was 45.91 ± 12.99 years, and the gender ratio was 1:0.87. The mean age of hypertension was 54.03 ± 11.37 years, and the gender ratio was 1:0.97. The prevalence of prehypertension and hypertension was significantly different within different age groups, gender, educational status, marital status, occupation, and family income per capita (all *p*-trend < 0.001). There was no difference in the prevalence between prehypertension and hypertension for different locations and ethnicity.

**Figure 1:**
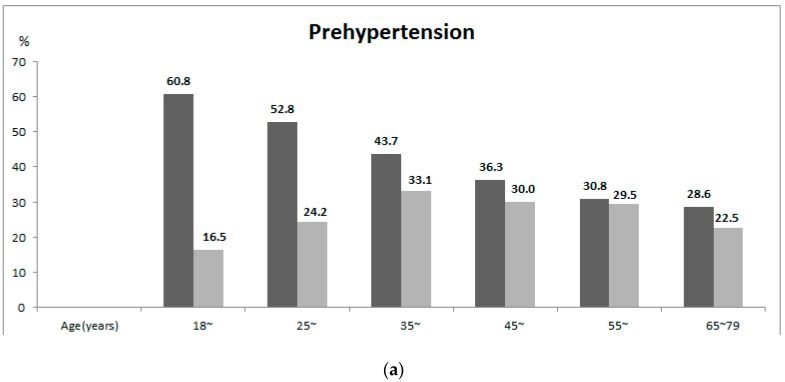

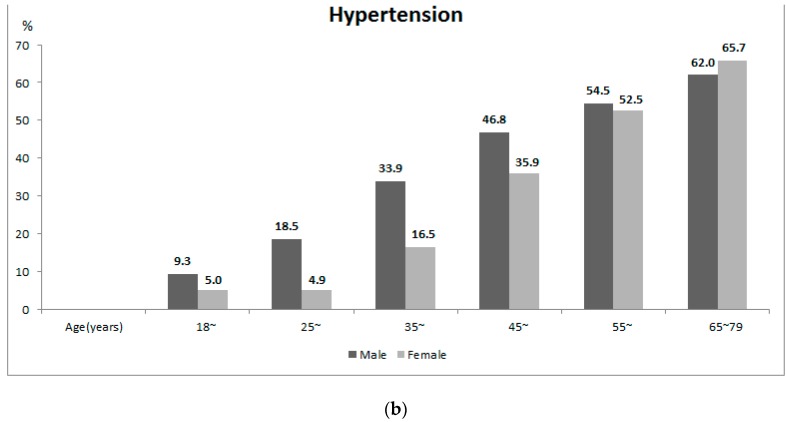
Age-specific prevalence of (**a**) prehypertension and (**b**) hypertension in in Jilin Province of China, 2012.

The age-specific prevalence of prehypertension and hypertension is listed in Figure 1. As age increased, the prevalence of prehypertension in males declined (*p*-trend < 0.001), although the prevalence of hypertension increased (*p*-trend < 0.001). The prevalence of prehypertension in females remained constant across younger age groups (<45 years) and decreased in older age groups. The prevalence of hypertension in females increased as age increased (*p*-trend < 0.001). The prevalence of prehypertension was 43.5% in men and 27.9% in women. The prevalence of hypertension was 34.9% in men and 26.4% in women.

**Table 1:** The prevalence of prehypertension and hypertension in the socio-demographic characteristics in Jilin Province of China, 2012 ^1^.

Variables	*N*	Prehypertension		Hypertension		χ^2^	*p*
		*n*	PR (95%*CI*)	*n*	PR (95%*CI*)		
Total	17,584	6036	36.0 (35.0–37.0)	6540	30.8 (29.9–31.6)		
Age (years)							
18–24	841	355	39.8 (35.3–44.5)	71	7.1 (5.2–9.7)	151.734	<0.001
25–34	2213	904	40.5 (37.8–43.1)	261	12.6 (10.8–14.7)		
35–44	3964	1498	38.5 (36.9–40.2)	955	25.4 (23.9–27.0)		
45–54	4822	1637	34.3 (32.9–35.7)	1977	41.4 (40.0–42.9)		
55–64	3919	1182	30.1 (28.6–31.7)	2099	53.5 (51.8–55.2)		
65–79	1825	460	25.5 (22.9–28.3)	1177	63.9 (60.9–66.7)		
Gender							
Female	9488	2808	27.9 (26.7–29.2)	3216	26.4 (25.3–27.5)	12.443	<0.001
Male	8096	3228	43.5 (42.0–45.0)	3324	34.9 (33.5–36.2)		
Location							
Urban	8944	3032	34.9 (33.6–36.2)	3140	30.4 (29.2–31.6)	0.542	0.462
Rural	8640	3004	37.3 (35.7–38.8)	3400	31.3 (30.0–32.6)		
Ethnicity							
Han	16,223	5565	35.9 (34.9–37.0)	6059	30.8 (29.9–31.8)	0.214	0.643
Other	1361	471	36.4 (32.8–40.2)	481	29.9 (26.7–33.3)		
Educational status (years)							
Primary school or below	5345	1719	34.0 (32.3–35.8)	2394	39.8 (38.1–41.5)	30.972	<0.001
Middle school	5010	1723	35.7 (33.8–37.5)	1875	31.4 (29.8–33.1)		
High school	4520	1613	37.2 (35.1–39.3)	1583	29.4 (27.8–31.1)		
Undergraduate and above	2709	981	37.0 (34.6–39.6)	688	20.9 (19.0–23.0)		
Marital status							
Married	15,136	5170	35.3 (34.3–36.3)	5744	32.9 (32.0–33.8)	37.802	<0.001
Unmarried	2448	866	38.6 (35.6–41.7)	796	22.2 (20.0–24.5)		
Occupation							
Manual	9962	3635	38.5 (37.2–39.9)	3479	29.0 (27.9–30.1)	87.142	<0.001
Brain	3363	1183	36.2 (33.8–38.6)	969	22.8 (21.1–24.6)		
Retired/unemployed	4259	1218	29.0 (27.2–31.0)	2092	44.3 (42.3–46.3)		
Family income per capita (¥)							
<500	3586	1174	34.8 (32.8–36.8)	1585	38.7 (36.8–40.6)	17.348	<0.001
500–	3683	1247	35.0 (32.8–37.2)	1449	33.7 (31.7–35.7)		
1000–	5868	1989	35.0 (33.3–36.7)	2082	29.4 (27.9–30.9)		
2000–	4447	1626	38.4 (36.4–40.4)	1424	26.4 (24.8–28.1)		

^1^ Complex weighted computation was used for all of the prevalence and statistical analysis.

Table 2 shows an initial indication of rural–urban differences in demographics. No significant rural–urban difference was observed in the gender proportions. Urban adult residents were somewhat younger and much better educated than rural adults. A larger Han population lived in rural areas. The mean family income *per capita* was significantly lower in rural areas than it was in urban areas, whereas the rate of marriage in rural areas was higher than that in urban areas. The proportions of drinking and fruit, egg and meat intake ≥2 times/week were slightly higher in urban areas. The distribution of family history and hypercholesterolemia varied in rural and urban regions, but the amount was greater in urban areas.

**Table 2:** Demographics and Correlates of Rural and Urban Adults in Jilin Province of China ^1^.

Variables	Rural, *n* (%) ^2^	Urban, *n* (%) ^2^	χ^2^	*p*
Total	8640 (100.0)	8944 (100.0)		
Age (years)			52.164	0.002
18–24	266 (14.0)	575 (13.3)		
25–34	817 (18.1)	1396 (22.1)		
35–44	1868 (24.0)	2096 (22.6)		
45–54	2532 (20.7)	2290 (20.0)		
55–64	2271 (15.2)	1648 (13.4)		
65–79	886 (8.0)	939 (8.6)		
Gender			2.905	0.229
Female	4723 (49.1)	4765 (47.8)		
Male	3917 (50.9)	4179 (52.2)		
Ethnicity			22.387	<0.001
Han	8072 (93.5)	8151 (91.6)		
Other	568 (6.5)	793 (8.4)		
Educational status			2895.816	<0.001
Primary school or below	4103 (39.3)	1242 (10.6)		
Middle school	2584 (34.2)	2426 (26.1)		
High school	1314 (16.4)	3206 (35.8)		
Undergraduate and above	639 (10.1)	2070 (27.5)		
Marital status			338.080	<0.001
Married	7853 (86.2)	7283 (75.0)		
Unmarried	787 (13.8)	1661 (25.0)		
Occupation			809.274	<0.001
Manual	5984 (67.2)	3978 (45.7)		
Brain	1187 (18.0)	2176 (27.9)		
Retired/unemployed	1469 (14.8)	2790 (26.4)		
Family income *per capita* (¥)			2385.274	<0.001
<500	2862 (26.6)	724 (6.6)		
500–	2353 (27.0)	1330 (12.6)		
1000–	2017 (25.8)	3851 (43.0)		
2000–	1408 (20.5)	3039 (37.8)		
Drinking			17.239	0.003
Yes	2504 (31.9)	2932 (34.9)		
No	6136 (68.1)	6012 (65.1)		
Salt intake			3.555	0.180
Excess	3283 (39.4)	3376 (38.0)		
Normal	5357 (60.6)	5568 (62.0)		
Nutrition intake				
Fruit			311.138	<0.001
≥2 times/week	3757 (45.3)	5245 (58.6)		
<2 times/week	4883 (54.7)	3699 (41.4)		
Egg			54.358	<0.001
≥2 times/week	4953 (56.0)	5674 (61.5)		
<2 times/week	3687 (44.0)	3270 (38.5)		
Meat			223.905	<0.001
≥2 times/week	2409 (31.2)	3528 (42.2)		
<2 times/week	6231 (68.8)	516 (57.8)		
Family history			41.433	<0.001
Yes	3930 (46.9)	4610 (51.7)		
No	4710 (53.1)	4334 (48.3)		
BMI			9.465	0.089
Normal	5198 (63.8)	5435 (61.8)		
Overweight	2869 (29.4)	2956 (31.6)		
Obesity	573 (6.8)	553 (6.6)		
Abdominal obesity			1.751	0.323
Yes	2858 (29.4)	2875 (30.3)		
No	5782 (70.6)	6069 (69.7)		
Hypertriglyceridemia			6.77	0.056
Yes	3713 (38.7)	3577 (36.7)		
No	4927 (61.3)	5367 (63.3)		
Hypercholesterolemia			14.248	0.004
Yes	3061 (29.0)	3130 (31.6)		
No	5579 (71.0)	5814 (68.4)		

^1^ Complex weighted computation was used in the statistical analysis; **^2^** Numbers are unweighted and percentages are weighted.

### 3.2. Correlates of Prehypertension and Hypertension

Univariate and multivariate logistic regression were performed using SPSS to assess significant determinants of prehypertension and hypertension. Prehypertension or hypertension served as the dichotomous outcome variable. Socio-demographic characteristics, lifestyle, family history, obesity, abdominal obesity, triglyceride, and total cholesterol were the independent predictor variables. Age, gender, and location were corrected based on complex weighted computation. The results of the univariate logistic regression are presented in Table 3. Univariate logistic regression showed that age, gender, location, educational status, occupation, family income per capita, drinking, salt intake, fruit, eggs or meat intake ≥2 times/week, BMI, abdominal obesity, triglyceride, and total cholesterol were all significantly associated with prehypertension status for the entire sample. Univariate logistic regression was used to assess the correlates of individuals with hypertension (hypertension = 1, normotension = 0). This analysis showed that age, gender, location, educational status, marital status, occupation, family income per capita, drinking, salt intake, fruit or eggs intake ≥2 times/week, family history of circulatory system disease, BMI, abdominal obesity, hypertriglyceridemia, and hypercholesterolemia were all significantly associated with hypertension.

**Table 3:** Odds ratios and 95% confidence intervals for correlates of prehypertension compared to normotension and hypertension compared to normotension (univariate logistic regression).

Characteristics	Prehypertension		Hypertension	
	OR (95% CI)	*p*	OR (95% CI)	*p*
Age (years)		<0.001		<0.001
18–24	1.000		1.000	
25–34	1.149 (0.914, 1.444)	0.233	2.003 (1.353, 2.963)	0.001
35–44	1.423 (1.150, 1.762)	0.001	5.237 (3.663, 7.488)	<0.001
45–54	1.881 (1.519, 2.328)	<0.001	12.682 (8.898, 18.077)	<0.001
55–64	2.449 (1.956, 3.067)	<0.001	24.263 (16.933, 34.767)	<0.001
65–79	3.193 (2.389, 4.269)	<0.001	44.597 (30.114, 66.046)	<0.001
Gender (Male/Female)	3.293 (2.952, 3.674)	<0.001	2.787 (2.513, 3.090)	<0.001
Location (Urban/Rural)	0.849 (0.760, 0.948)	0.004	0.879 (0.793, 0.975)	0.015
Ethnicity (Other/Han)	0.998 (0.820, 1.215)	0.984	0.955 (0.788, 1.157)	0.637
Educational status (years)		<0.001		<0.001
Primary school or below	1.000		1.000	
Middle school	0.833 (0.721, 0.963)	0.013	0.628 (0.548, 0.719)	<0.001
High school	0.857 (0.739, 0.995)	0.043	0.580 (0.506, 0.665)	<0.001
Undergraduate and above	0.678 (0.579, 0.794)	<0.001	0.327 (0.278, 0.385)	<0.001
Marital status (Unmarried/Married)	0.885 (0.757, 1.036)	0.129	0.617 (0.528, 0.721)	<0.001
Occupation		<0.001		<0.001
Manual	1.000		1.000	
Brain	0.743 (0.649, 0.852)	<0.001	0.624 (0.546, 0.712)	<0.001
Retired/unemployed	0.918 (0.796, 1.059)	0.240	1.863 (1.636, 2.122)	<0.001
Family income *per capita* (¥)		0.002		<0.001
<500	1.000		1.000	
500–	0.850 (0.717,1.008)	0.062	0.735 (0.629,0.859)	<0.001
1000–	0.750 (0.646,0.871)	<0.001	0.567 (0.494,0.651)	<0.001
2000–	0.832 (0.714,0.970)	0.019	0.514 (0.446,0.594)	<0.001
Drinking (Yes/No)	1.963 (1.746,2.208)	<0.001	2.030 (1.816,2.270)	<0.001
Salt intake (Excess/Normal)	1.140 (1.020,1.273)	0.021	1.247 (1.124,1.384)	<0.001
Nutrition intake ^1^				
Fruit ^1^: ≥ 2 times/week	0.752 (0.675,0.839)	<0.001	0.589 (0.531,0.652)	<0.001
Egg ^1^: ≥ 2 times/week	1.297 (1.162,1.448)	<0.001	1.397 (1.259,1.550)	<0.001
Meet ^1^: ≥ 2 times/weeek	1.199 (1.072,1.340)	<0.001	0.963 (0.864,0.072)	0.486
Family history (Yes/No)	1.010 (0.906,1.125)	0.858	1.290 (1.165,1.428)	<0.001
BMI		<0.001		<0.001
Normal	1.000		1.000	
Overweight	2.217 (1.960,2.508)	<0.001	4.552 (4.042,5.125)	<0.001
Obesity	3.923 (2.829,5.440)	<0.001	8.492 (6.307,11.435)	<0.001
Abdominal obesity (Yes/No)	2.422 (2.114,2.775)	<0.001	5.440 (4.789,6.181)	<0.001
Hypertriglyceridemia (Yes/No)	2.176 (1.939,2.442)	<0.001	4.531 (4.062,5.054)	<0.001
Hypercholesterolemia (Yes/No)	1.828 (1.631,2.050)	<0.001	3.473 (3.119,3.867)	<0.001

^1^ The reference for each type of nutrition intake was frequency <2 times/week.

Multivariate logistic regression analysis showed that age, gender, location, drinking, eggs intake ≥2 times/week, BMI, abdominal obesity, hypertriglyceridemia, and hypercholesterolemia are significantly associated with prehypertension (Table 4). Compared with people aged 18–24 years, people aged 45–54 years, 55–64 years and ≥65 years had a greater correlation of developing prehypertension (OR = 1.538, 1.935, 2.638, respectively). Male participants were more likely to develop prehypertension than females (OR = 3.085). For prehypertension, those living in urban areas were associated with a lower prevalence of prehypertension (OR = 0.828). Drinkers had a greater correlation of developing prehypertension (OR = 1.175). People with a higher eggs intake were 1.188 times more likely to develop prehypertension than others were. People with a status of overweight or obese were associated with a higher prevalence of prehypertension compared with people of normal weight (25 ≤ BMI < 30, OR = 1.655; BMI ≥ 30, OR = 2.960). Participants with abdominal obesity had a greater correlation of developing prehypertension (OR = 1.228). Both hypertriglyceridemia and hypercholesterolemia were associated with a greater likelihood of prehypertension (OR = 1.133, 1.110).

Determinants of hypertension (*vs.* normotension) via multivariable logistic regression analysis were shown in Table 4. A clear trend toward a greater correlation of hypertension was noted as age increased. Starting from 35 years, every 10 years increment in age produced a 2.833 times, 6.936 times, 14.729 times, and 34.613 times greater likelihood of hypertension than their respective decadal reference group (aged 18–24 years). Males were positively associated with hypertension. Compared to females, males were more likely to develop hypertension (OR = 2.383). Drinkers had a greater correlation of developing hypertension (OR = 1.629). Fruit intake ≥2 times/week was shown to be less likely to develop hypertension (OR = 0.780). People with hypertension more frequently had a family history of circulatory system disease (OR = 1.604). Overweight (OR = 2.676) and obesity (OR = 5.716) were the most significant determinants of hypertension. Our findings showed that abdominally obese subjects had a 1.537 times greater correlation of hypertension compared with normal weight subjects. Both hypertriglyceridemia and hypercholesterolemia were associated with a greater likelihood of hypertension (OR = 2.015, 1.377).

**Table 4:** Odds ratios and 95% confidence intervals for correlates of prehypertension compared to normotension and hypertension compared to normotension (multivariate logistic regression) ^1^.

Characteristics	Prehypertension		Hypertension	
	OR (95% CI)	*p*	OR (95% CI)	*p*
Age (years)		<0.001		<0.001
18–24	1.000		1.000	
25–34	0.904 (0.718, 1.139)	0.394	1.112 (0.700, 1.764)	0.653
35–44	1.239 (0.994, 1.544)	0.057	2.833 (1.843, 4.354)	<0.001
45–54	1.538 (1.228, 1.926)	<0.001	6.936 (4.496, 10.701)	<0.001
55–64	1.935 (1.518, 2.467)	<0.001	14.729 (9.450, 22.957)	<0.001
65–79	2.638 (1.952, 3.565)	<0.001	34.613 (21.475, 55.790)	<0.001
Gender (Male/Female)	3.085 (2.711, 3.510)	<0.001	2.383 (2.073, 2.739)	<0.001
Location (Urban/Rural)	0.828 (0.738, 0.929)	0.001	0.886 (0.777, 1.010)	0.070
Drinking (Yes/No)	1.175 (1.022, 1.351)	0.024	1.629 (1.389, 1.910)	<0.001
Fruit ^2^:≥2times/week	–	–	0.780 (0.683, 0.890)	<0.001
Egg ^2^:≥2times/week	1.188 (1.057, 1.334)	0.004	–	–
Family history (Yes/No)	–	–	1.604 (1.407, 1.829)	<0.001
BMI		<0.001		<0.001
Normal	1.000		1.000	
Overweight	1.665 (1.428, 1.919)	<0.001	2.676 (2.295, 3.121)	<0.001
Obesity	2.960 (2.045, 4.285)	<0.001	5.716 (3.983, 8.203)	<0.001
Abdominal obesity (Yes/No)	1.228 (1.031, 1.462)	0.021	1.537 (1.299, 1.818)	<0.001
Hypertriglyceridemia (Yes/No)	1.301 (1.133, 1.493)	<0.001	2.015 (1.748, 2.324)	<0.001
Hypercholesterolemia (Yes/No)	1.261 (1.110, 1.432)	<0.001	1.377 (1.198, 1.582)	<0.001

^1^ “–“: The variable did not enter the model.

## 4. Discussion

The present study data for prehypertension and hypertension were derived from a stratified, clustered multistage and random survey sample of people aged 18–79 years in the Jilin Province and were analyzed based on a complex sampling model.

Our findings show that prehypertension affects about one third (36.0%) of adults in the Jilin Province in northeastern China. This is generally consistent with published studies, such as the figures of 36.1% in Brazilian adults [10] and 33.7% among adults in southern Iran [11]. Compared with other studies within China [12,13], although the prevalence of prehypertension in this study was lower than that in inner Mongolian adults (38.4%); and higher than that in Taiwanese adults (34.0%), the rate was comparable with that in Beijing adults (35.7%).

Prehypertension and hypertension, which accounted for two thirds of the total population, appeared to be quite common in northeastern China. Furthermore, in the present study, we found that the prevalence of prehypertension decreased in males as age increased; and decreased in females in those aged ≥35 years, which was concordant with Liaoning Province adults [14]. In the current study, individuals aged 45–79 years were less likely to have prehypertension than younger individuals were. This was because most of the individuals in the older age groups had progressed to actual hypertension, similar to other studies [15]. Therefore, the highest prevalence of hypertension was in the 65–79 age group for men (62.0%) and women (65.7%).

Another finding of the present study was that the prevalence of hypertension increased with age in both genders and that this increase was more rapid in females than males. Consistent with previous reports, the rates of hypertension were higher in men than in women at younger ages, whereas the reverse was true in older participants [15]. The specific reason for this difference may be related to survival bias or hormonal changes at different ages in the two genders, but this required further study. Consistent with this, the multivariate logistic regression analysis showed a significant increase in the ORs for males in the prehypertension (OR = 3.085) and hypertension (OR = 2.383) groups.

In this study, living in an urban area led to a reduced correlation of prehypertension (OR = 0.828) (Table 4). The influence of location as an independent associated factor for prehypertension and hypertension has rarely been mentioned in other studies. Individual studies [13] have reported the different risk factors associated with prehypertension and hypertension among residents in urban or rural locations. The difference in environment and lifestyle in urban or rural locations may underlie this result. According to our research on demographics and correlates of rural and urban adults in Table 2, urban residents were somewhat younger than rural residents, but they are better educated and have higher income. The rates of drinking and fruit, egg and meat intake ≥2 times/week were slightly higher in urban areas. Rural populations may lack information and knowledge about health, and a lower level of education among rural adults compared with urban adults may lead to unhealthy lifestyles and unawareness about the prevention of prehypertension and hypertension. Vigorous healthy males from rural families may leave the village to work in urban areas, leaving elders in rural areas, which may be harmful to the health of those who remain. The explanation for this observation requires further investigation.

In our study, drinking was associated not only with hypertension but also with prehypertension (Table 4). The pathology of drinking-related elevated blood pressure was through the effects of alcohol on neural conduction, blood vessels, plasma vasopressin, cardiac function, noradrenaline metabolism, acetaldehyde, sympathetic activity, the renin–angiotensin system and adreno-corticotropic hormone, and calcium metabolism [16].

In particular, high egg intake (≥2 times/week) was an important factor associated with prehypertension but not for hypertension. Interestingly, we found a high prevalence of prehypertension in individuals with a high egg intake. However, this result was not comparable with other studies because the standard of egg intake was not the same [17]. Hypertensive patients might have the same frequency of egg intake but consume fewer eggs than they did before they knew they had hypertension.This would result in reduced egg intake, and egg intake would thus not be an independent factor. Therefore, to avoid increased blood pressure, egg intake should also be controlled.

However, a low fruit intake (<2 times/week) and a family history of circulatory system disease were significantly associated with hypertension but not prehypertension (Table 4). In this study, hypertension had a strong inverse association with the frequency of fruit intake. Previous observational studies have reported that a high fruit intake is associated with a reduced risk of developing cardiovascular disease and stroke, as well as decreased blood pressure [18]. The probable reason why a high fruit intake is positively associated with hypertension is that fruits contain many vitamins and minerals whose metabolites act on the vascular endothelium and lower blood pressure [18]. Thus, it is important for everyone to increase their fruit intake, particularly those with hypertension. We found an important correlation between a family history of circulatory system disease and hypertension. The normotensive individuals with a family history of circulatory system disease were 1.604 times more likely to have hypertension than those without such a family history (Table 4). Therefore, primary prevention strategies should be targeted at prehypertensive individuals who have a family history of circulatory system disease.

In this study, obesity was identified as being more associated with prehypertension (OR = 2.960) than with hypertension (OR = 5.716). It is worth noting that obesity had the highest correlation with hypertension among the normotensive individuals apart from age (Table 4). When obesity coexists with prehypertension, it may further increase the correlation of developing hypertension. Many studies have reported that waist circumference is associated with a higher prevalence of prehypertension and hypertension [19]. Abdominal obesity was also found to be associated with increased blood pressure according to adjusted BMI, triglycerides, and cholesterol (Table 4).

In another study [20], there was a significant association between prehypertension or hypertension and hypertriglyceridemia or hypercholesterolemia. In our current study, hypertriglyceridemia and hypercholesterolemia were both significantly associated with prehypertension and/or hypertension. Meanwhile, those with hypertriglyceridemia had a 2.015 times greater correlation of hypertension compared with the normal group (Table 4).

This study has several limitations. The first limitation was the low response rate, which often exists in cross-sectional epidemiological studies. Second, our data were obtained from a cross-sectional study in the Jilin Province and were not representative of adults throughout China. Third, because this is a cross-sectional study, the interpretation of causal relationships between risk factors and the development of prehypertension and hypertension is limited. Fourth, during our survey, we created analogous “clinic environments” by assembling participants for anthropometric examinations. The higher blood pressures as observed in our study participants might be explained in part as a result of the white-coat effect that might be associated with the clinic environments in which these blood pressures were measured [21,22]. Furthermore, due to the limitation of economy and human resource, there was no objective measurement to assess the “salt intake”.

Despite the above limitations, the results of this study as community-based data could be important in providing relevant epidemiological information to help promote health for the primary prevention of hypertension. We used the complex weighted computation by adjusting for age, gender, and location to approximate the actual population composition of the Jilin Province in northeastern China. This study reinforced the need to urgently address the problem of prehypertension and hypertension in China by establishing an effective health security system for the public.

## 5. Conclusions

Prehypertension and hypertension are highly prevalent in northeastern China. Prevention and treatment are urgently needed to address the public health problem of prehypertension and hypertension and to prevent prehypertensive people from developing hypertension or cardiovascular disease. For individuals with normotension, these common correlates of both prehypertension and hypertension (rural, drinking, overweight, obesity, abdominal obesity, hypertriglyceridemia, and hypercholesterolemia) should be controlled as the most important factors. In addition, our results are the first to suggest that living in an urban environment is associated with a lower prevalence of prehypertension. These factors are probably important in decreasing the risk of developing hypertension among prehypertensive and normotensive people.

## References

[1] WHO Global Status Report on Noncommunicable Diseases 2014. http://apps.who.int/iris/bitstream/10665/148114/1/9789241564854_eng.pdf?ua=1.

[2] Danaei G., Finucane M.M., Lin J.K., Singh G.M., Paciorek C.J., Cowan M.J., Farzadfar F., Stevens G.A., Lim S.S., Riley L.M. (2011). National, regional, and global trends in systolic blood pressure since 1980: Systematic analysis of health examination surveys and epidemiological studies with 786 country-years and 5.4 million participants. Lancet.

[3] Kearney P.M., Whelton M., Reynolds K., Muntner P., Whelton P.K., He J. (2005). Global burden of hypertension: Analysis of worldwide data. Lancet.

[4] Lenfant C., Chobanian A.V., Jones D.W., Roccella E.J. (2003). Seventh report of the joint national committee on the prevention, detection, evaluation, and treatment of high blood pressure (JNC 7): Resetting the hypertension sails. Hypertension.

[5] Vasan R.S., Larson M.G., Leip E.P., Evans J.C., O’Donnell C.J., Kannel W.B., Levy D. (2001). Impact of high-normal blood pressure on the risk of cardiovascular disease. N. Engl. J. Med..

[6] Vasan R.S., Larson M.G., Leip E.P., Kannel W.B., Levy D. (2001). Assessment of frequency of progression to hypertension in non-hypertensive participants in the framingham heart study: A cohort study. Lancet.

[7] Suri M.F., Qureshi A.I. (2006). Prehypertension as a risk factor for cardiovascular diseases. J. Cardiovasc. Nurs..

[8] Li Z., Yao Y., Han W., Yu Y., Liu Y., Tao Y., Kou C., Jiang L., Sun Q., Yin Y. (2015). Smoking prevalence and associated factors as well as attitudes and perceptions towards tobacco control in northeast China. Int. J. Environ. Res. Public Health.

[9] National Cholesterol Education Program (NCEP) Expert Panel (2002). Third report of the national cholesterol education program (NCEP) expert panel on detection, evaluation, and treatment of high blood cholesterol in adults (adult treatment panel III) final report. Circulation.

[10] Silva D.A., Petroski E.L., Peres M.A. (2012). Prehypertension and hypertension among adults in a metropolitan area in southern brazil: Population-based study. Rev. Saude Publica.

[11] Rahmanian K., Shojaie M. (2012). The prevalence of pre-hypertension and its association to established cardiovascular risk factors in south of Iran. BMC Res. Notes.

[12] Li H., Xu T., Tong W., Liu Y., Zhao L., Zhang Y. (2008). Comparison of cardiovascular risk factors between prehypertension and hypertension in a Mongolian population, Inner Mongolia, China. Circ. J..

[13] Zhang W.H., Zhang L., An W.F., Ma J.L. (2011). Prehypertension and clustering of cardiovascular risk factors among adults in suburban Beijing, China. J. Epidemiol..

[14] Sun Z., Zheng L., Wei Y., Li J., Zhang X., Zhang X., Liu S., Xu C., Li J., Zhao F. (2007). Prevalence and risk factors of the rural adult people prehypertension status in Liaoning Province of China. Circ. J..

[15] Guo X., Zheng L., Li Y., Yu S., Zhou X., Wang R., Zhang X., Sun Z., Sun Y. (2013). Gender-specific prevalence and associated risk factors of prehypertension among rural children and adolescents in Northeast China: A cross-sectional study. Eur. J. Pediatr..

[16] Okubo Y., Sairenchi T., Irie F., Yamagishi K., Iso H., Watanabe H., Muto T., Tanaka K., Ota H. (2014). Association of alcohol consumption with incident hypertension among middle-aged and older Japanese population: The ibarakai prefectural health study (IPHS). Hypertension.

[17] Pitsavos C., Milias G.A., Panagiotakos D.B., Xenaki D., Panagopoulos G., Stefanadis C. (2006). Prevalence of self-reported hypertension and its relation to dietary habits, in adults; a nutrition & health survey in Greece. BMC Public Health.

[18] Miura K., Greenland P., Stamler J., Liu K., Daviglus M.L., Nakagawa H. (2004). Relation of vegetable, fruit, and meat intake to 7-year blood pressure change in middle-aged men: The Chicago western electric study. Am. J. Epidemiol..

[19] Dong G.H., Wang D., Liu M.M., Liu Y.Q., Zhao Y., Yang M., Meng X.J., Tian S., Meng X., Zhang H.Y. (2012). Sex difference of the prevalence and risk factors associated with prehypertension among urban Chinese adults from 33 communities of China: The CHPSNE study. J. Hypertens..

[20] Lin Y., Lai X., Chen G., Xu Y., Huang B., Chen Z., Zhu S., Yao J., Jiang Q., Huang H. (2012). Prevalence and risk factors associated with prehypertension and hypertension in the Chinese She population. Kidney Blood Press. Res..

[21] Sung S.H., Cheng H.M., Wang K.L., Yu W.C., Chuang S.Y., Ting C.T., Lakatta E.G., Yin F.C., Chou P., Chen C.H. (2013). White coat hypertension is more risky than prehypertension: Important role of arterial wave reflections. Hypertension.

[22] Agarwal R., Weir M.R. (2013). Treated hypertension and the white coat phenomenon: Office readings are inadequate measures of efficacy. J. Am. Soc. Hypertens..

